# Supplementary material for: Single-cell transcriptomics reveals striking heterogeneity and functional organization of dendritic and monocytic cells in the bovine mesenteric lymph node
Source: Front Immunol. 2023 Jan 6;13:1099357. doi: 10.3389/fimmu.2022.1099357 (PMC9853064; doi:10.3389/fimmu.2022.1099357)
Supplement: Supplementary file 1 [file DataSheet_1.pdf]

Supplementary File 1A

MLN2306

The analysis detected some issues. Details »

Alert	Value	Detail
⚠ **Low Fraction Valid UMIs**	69.4%	Ideal > 75%. This may indicate a quality issue with the Illumina R2 read for Single Cell 3' v1 or the R1 read for Single Cell 3' v2/v3 and Single Cell 5'. Application performance may be affected.

Estimated Number of Cells
6,937

Mean Reads per Cell	Median Genes per Cell
120,798	898

Sequencing

Number of Reads	837,977,792
Valid Barcodes	97.1%
Sequencing Saturation	61.7%
Q30 Bases in Barcode	96.5%
Q30 Bases in RNA Read	94.1%
Q30 Bases in UMI	96.4%

Mapping

Reads Mapped to Genome	95.7%
Reads Mapped Confidently to Genome	88.5%
Reads Mapped Confidently to Intergenic Regions	11.5%
Reads Mapped Confidently to Intronic Regions	19.5%
Reads Mapped Confidently to Exonic Regions	57.5%
Reads Mapped Confidently to Transcriptome	54.3%
Reads Mapped Antisense to Gene	1.1%

Cells

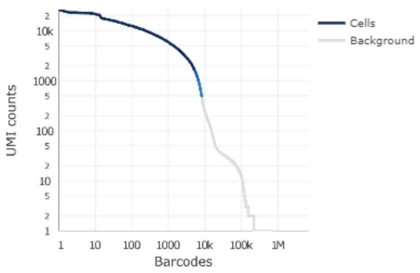

Estimated Number of Cells	6,937
Fraction Reads in Cells	88.0%
Mean Reads per Cell	120,798
Median Genes per Cell	898
Total Genes Detected	14,493
Median UMI Counts per Cell	2,685

Sample

Name	MLN2306
Description	
Transcriptome	Bos_taurus.ARS-UCD1.2.dna.toplevel
Chemistry	Single Cell 3' v3
Cell Ranger Version	3.0.2

MLN3006

The analysis detected some issues. Details »

Alert	Value	Detail
⚠ **Low Fraction Valid UMIs**	69.8%	Ideal > 75%. This may indicate a quality issue with the Illumina R2 read for Single Cell 3' v1 or the R1 read for Single Cell 3' v2/v3 and Single Cell 5'. Application performance may be affected.

Estimated Number of Cells
5,187

Mean Reads per Cell	Median Genes per Cell
147,824	2,007

Sequencing

Number of Reads	766,767,159
Valid Barcodes	96.6%
Sequencing Saturation	44.0%
Q30 Bases in Barcode	96.3%
Q30 Bases in RNA Read	93.9%
Q30 Bases in UMI	96.2%

Mapping

Reads Mapped to Genome	84.3%
Reads Mapped Confidently to Genome	77.3%
Reads Mapped Confidently to Intergenic Regions	16.9%
Reads Mapped Confidently to Intronic Regions	26.2%
Reads Mapped Confidently to Exonic Regions	34.2%
Reads Mapped Confidently to Transcriptome	30.7%
Reads Mapped Antisense to Gene	1.5%

Cells

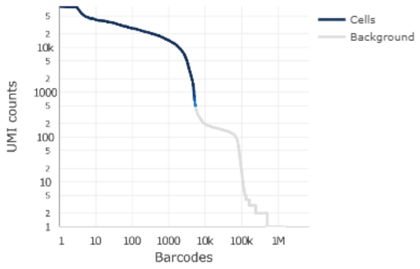

Estimated Number of Cells	5,187
Fraction Reads in Cells	79.1%
Mean Reads per Cell	147,824
Median Genes per Cell	2,007
Total Genes Detected	15,844
Median UMI Counts per Cell	7,157

Sample

Name	MLN3006
Description	
Transcriptome	Bos_taurus.ARS-UCD1.2.dna.toplevel
Chemistry	Single Cell 3' v3
Cell Ranger Version	3.0.2

Cell Ranger · MLN0707 ·

SUMMARY ANALYSIS

MLN0707

The analysis detected some issues. Details »

Alert	Value	Detail
Low Fraction Valid UMIs	69.8%	Ideal > 75%. This may indicate a quality issue with the Illumina R2 read for Single Cell 3' v1 or the R1 read for Single Cell 3' v2/v3 and Single Cell 5'. Application performance may be affected.

Estimated Number of Cells

4,288

Mean Reads per Cell	Median Genes per Cell
176,752	2,155

Sequencing

Number of Reads	757,914,774
Valid Barcodes	96.5%
Sequencing Saturation	49.9%
Q30 Bases in Barcode	96.3%
Q30 Bases in RNA Read	94.1%
Q30 Bases in UMI	96.2%

Mapping

Reads Mapped to Genome	85.8%
Reads Mapped Confidently to Genome	79.6%
Reads Mapped Confidently to Intergenic Regions	16.7%
Reads Mapped Confidently to Intronic Regions	27.5%
Reads Mapped Confidently to Exonic Regions	35.4%
Reads Mapped Confidently to Transcriptome	32.0%
Reads Mapped Antisense to Gene	1.5%

Cells

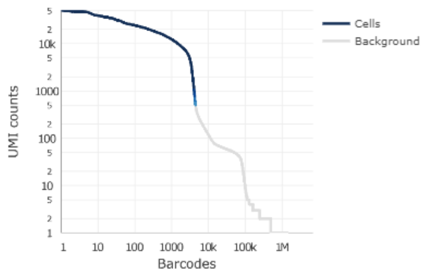

Estimated Number of Cells	4,288
Fraction Reads in Cells	86.9%
Mean Reads per Cell	176,752
Median Genes per Cell	2,155
Total Genes Detected	15,713
Median UMI Counts per Cell	7,819

Sample

Name	MLN0707
Description	
Transcriptome	Bos_taurus.ARS-UCD1.2.dna.toplevel
Chemistry	Single Cell 3' v3
Cell Ranger Version	3.0.2

Supplementary File 1A Cell Ranger summaries for the three samples (MLN2306, MLN3006, MLN0707) analyzed by 10x scRNA-seq.

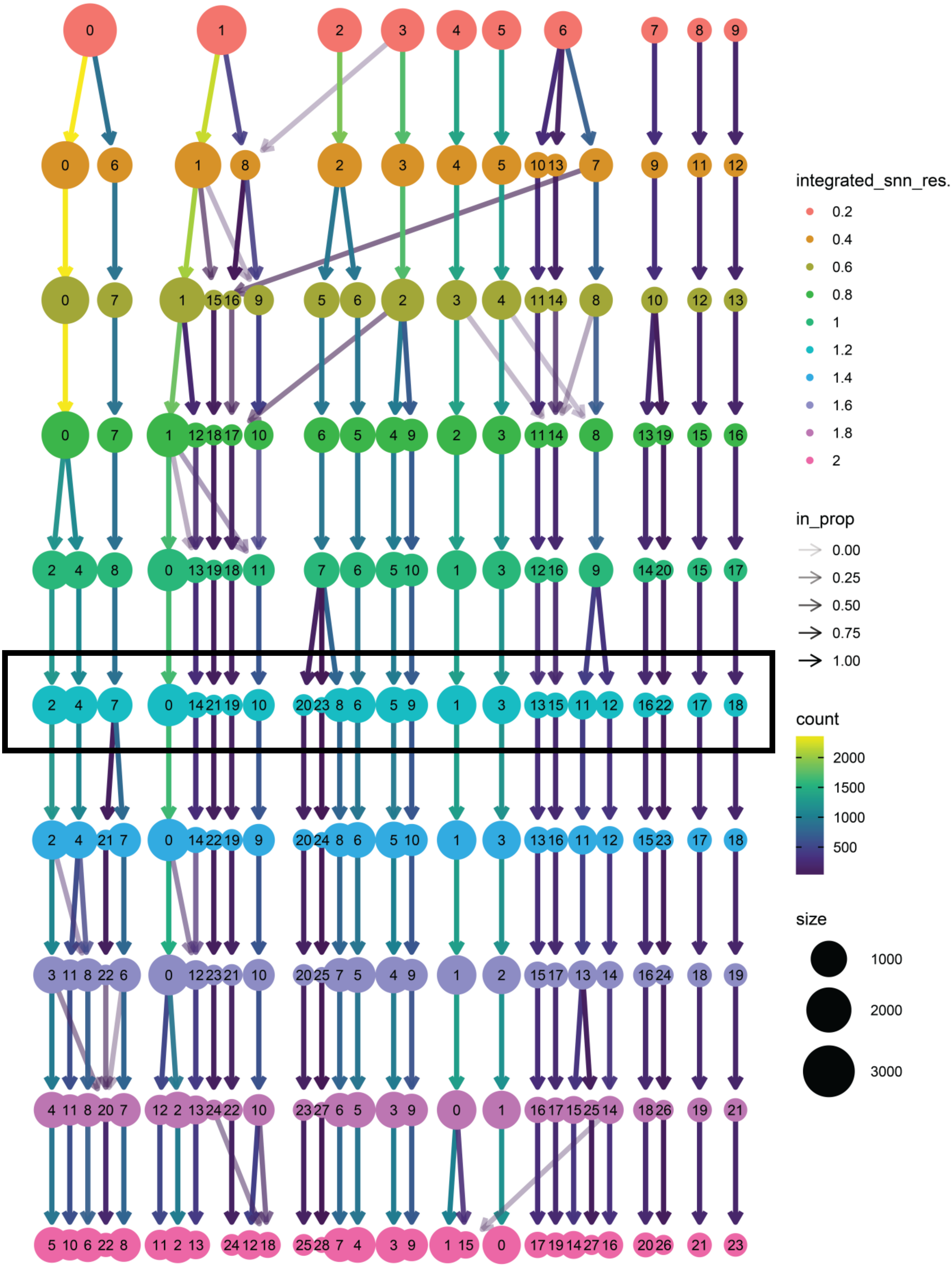

Supplementary File 1B Evaluation of cluster stability (integrated dataset) using the clustree package (Zappia et al. 2018). A resolution of 1.2 was chosen for downstream analyses, resulting in 24 different clusters.

Supplementary File 1C-F

C Individual samples before data integration (colors not matched)

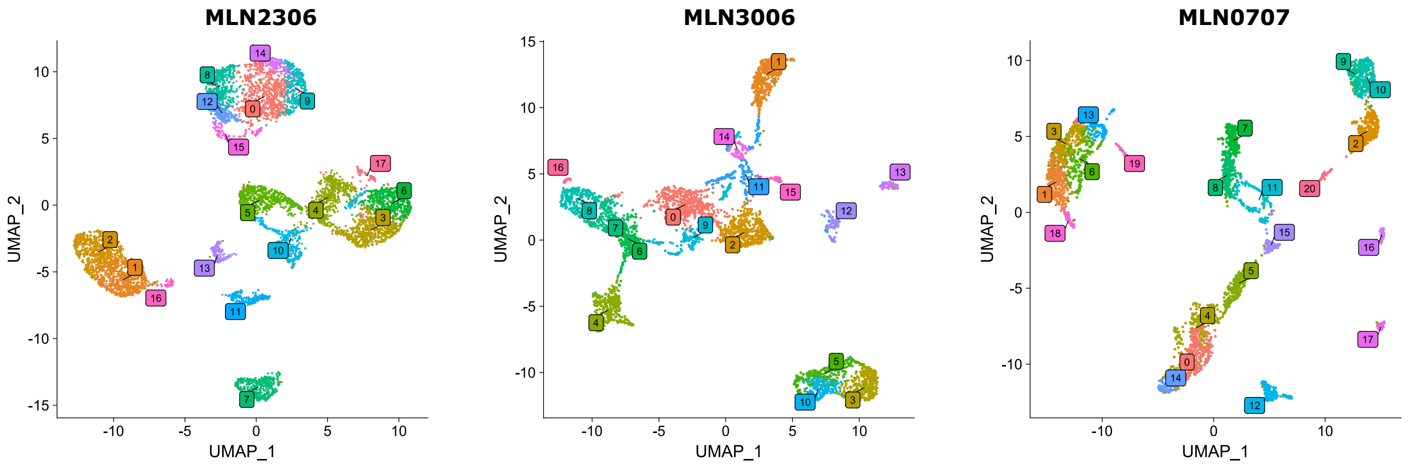

D Individual samples after data integration

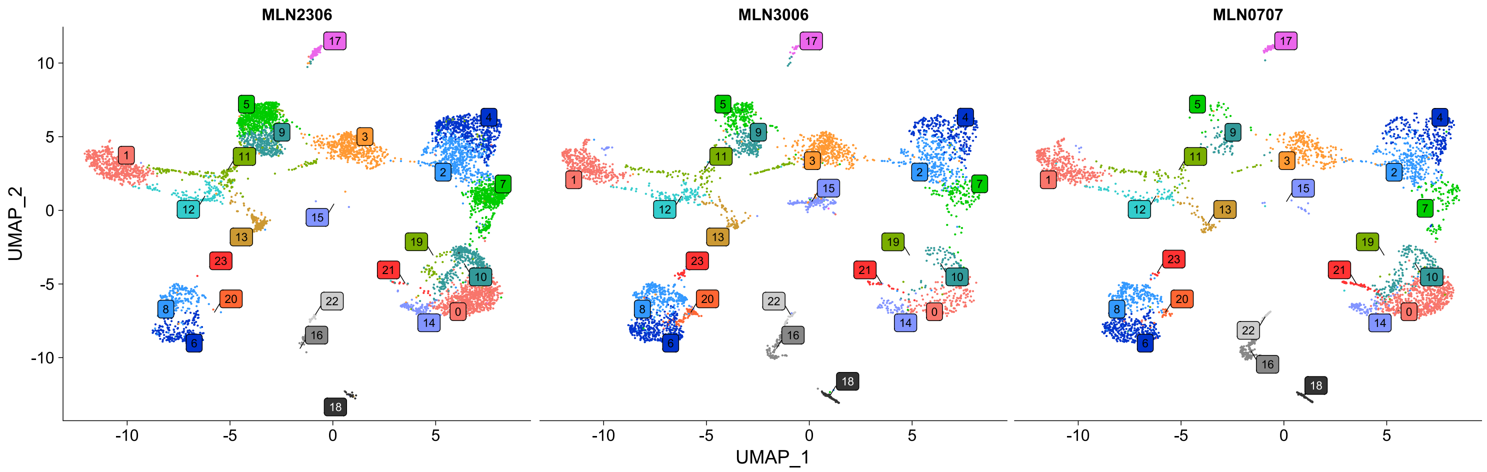

E Integrated data

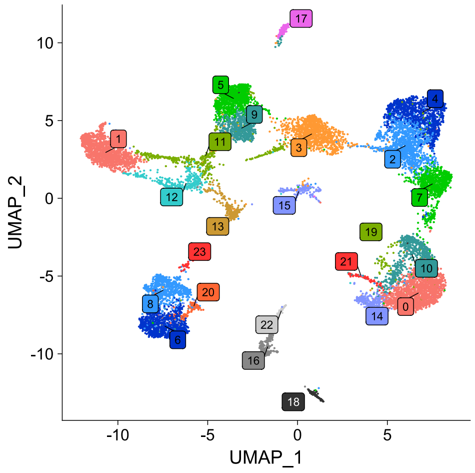

F Cluster composition per sample

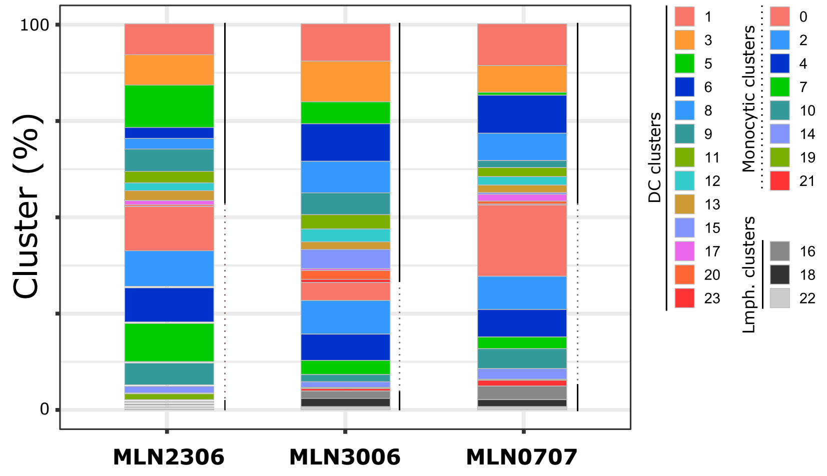

Supplementary File 1C-F Individual samples before and after data integration. (C) Non-integrated datasets. Clustering was performed at a resolution of 1.2. Differentially expressed genes for each dataset are given in Supplementary File 2. (D) Integrated dataset split by samples. (E) Complete integrated dataset. Clustering was performed at a resolution of 1.2. (F) Proportional distribution of clusters in each sample after data integration. Lines on the side of each bar indicate clusters assigned as dendritic cells, monocytic cells, and lymphocytes. Detailed cluster assignments are given in Figure 1B.

Supplementary File 1G (page 1/2)

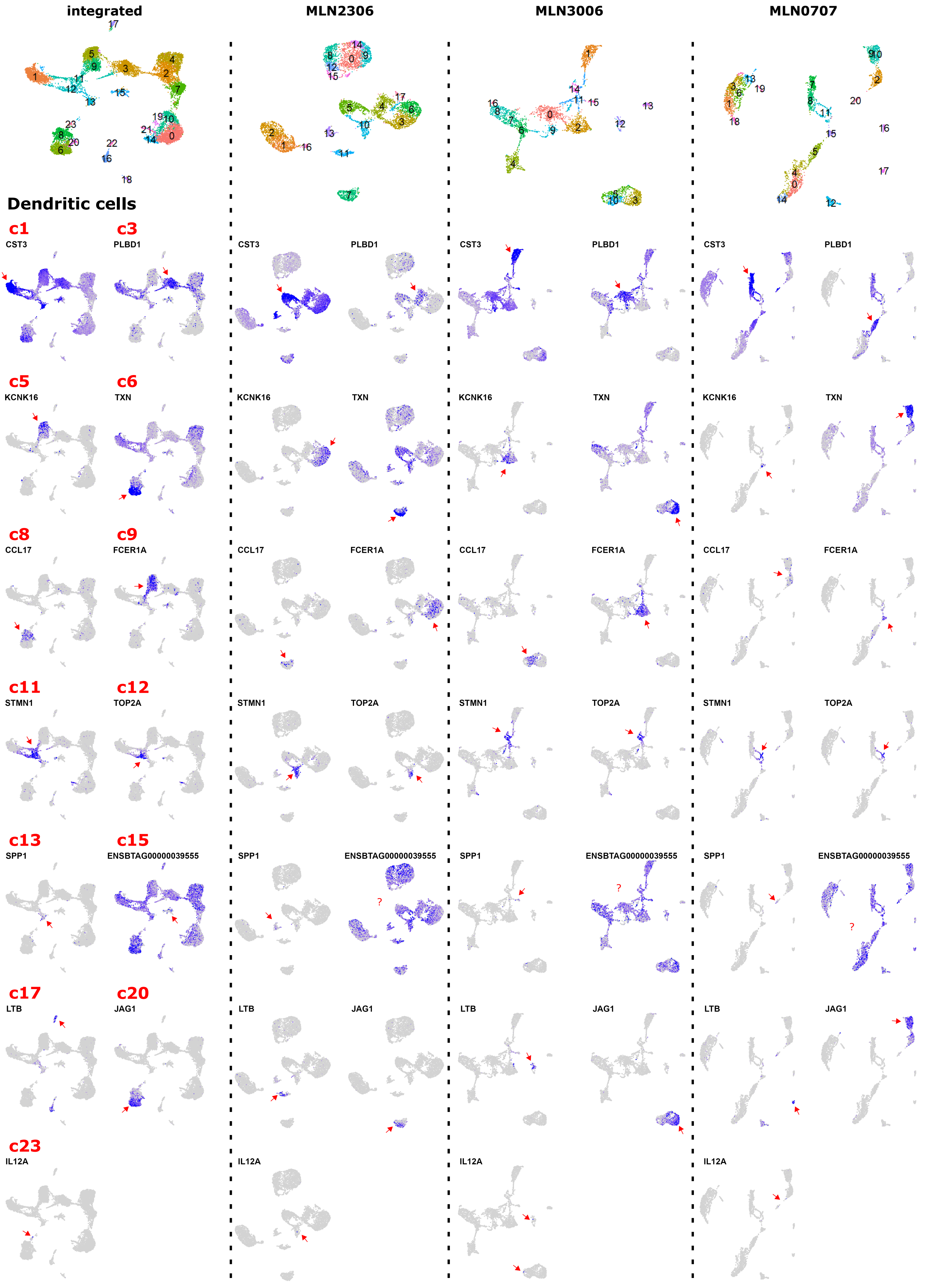

Supplementary File 1G (page 2/2)

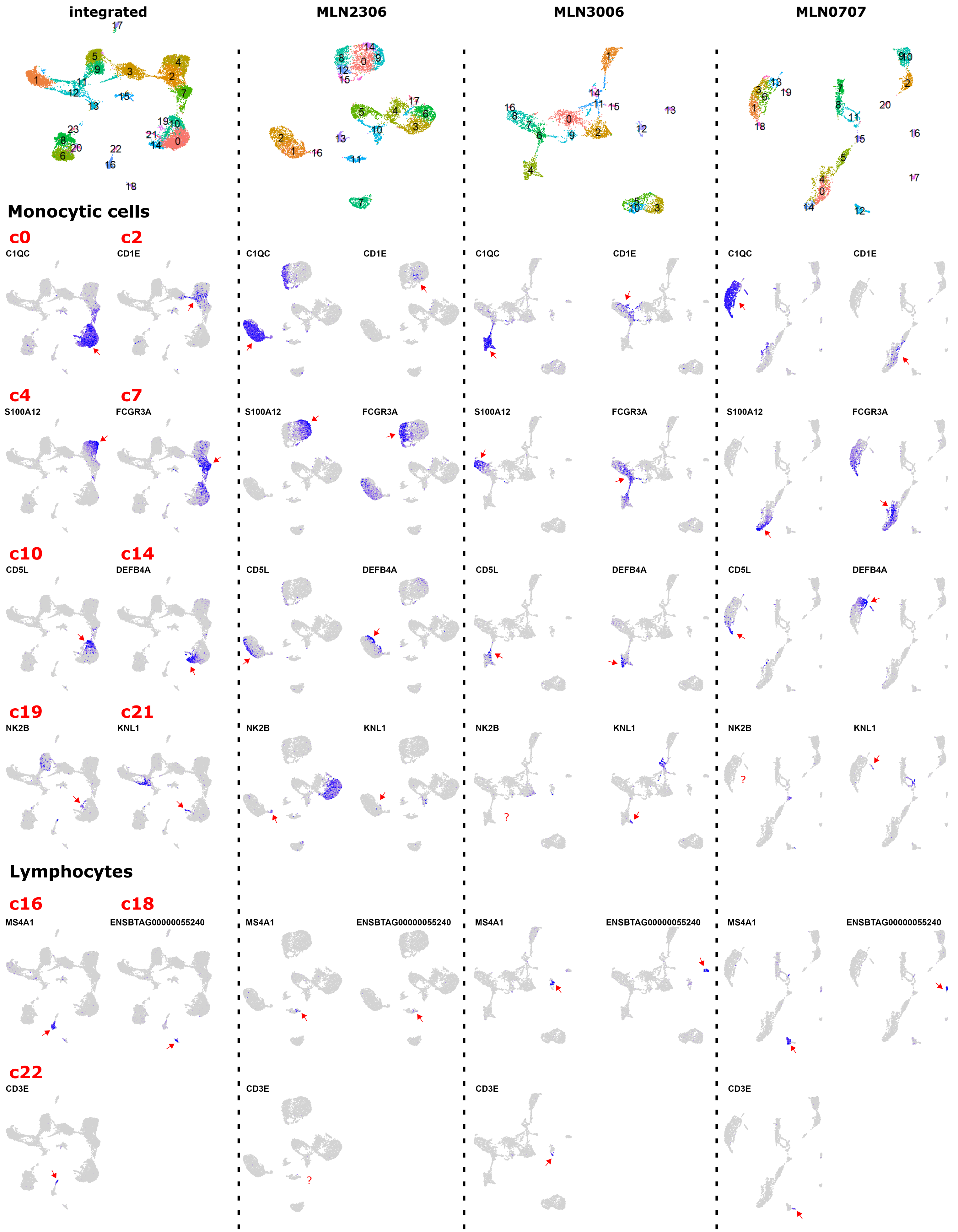

Supplementary File 1G Visualization of analogous clusters in non-integrated datasets. For each cluster of the integrated dataset the top marker gene (FindAllMarkers, lowest p_val_adj followed by highest avg_log2FC) was visualized for the three non-integrated datasets. The order of clusters follows their categorization into dendritic cells, monocytic cells and lymphocytes (see also Figure 1D). Detailed cluster assignments are given in Figure 1B. Differentially expressed genes for each dataset are given in Supplementary File 2.
