# Supplementary material for: Single-cell transcriptomics reveals striking heterogeneity and functional organization of dendritic and monocytic cells in the bovine mesenteric lymph node
Source: Front Immunol. 2023 Jan 6;13:1099357. doi: 10.3389/fimmu.2022.1099357 (PMC9853064; doi:10.3389/fimmu.2022.1099357)
Supplement: Supplementary file 5 [file DataSheet_5.pdf]

Supplementary File 5

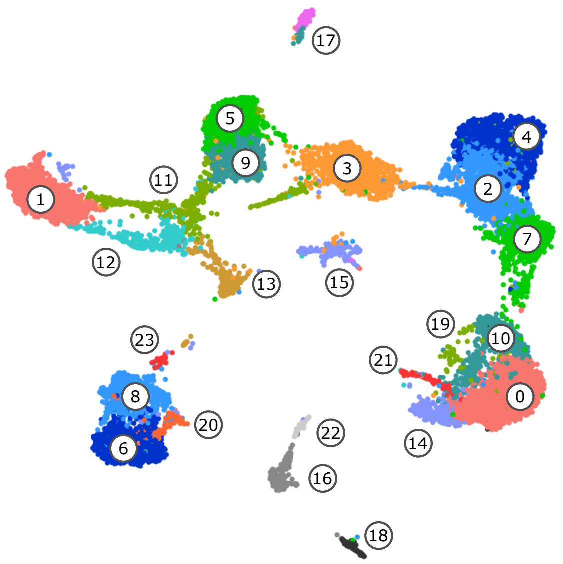

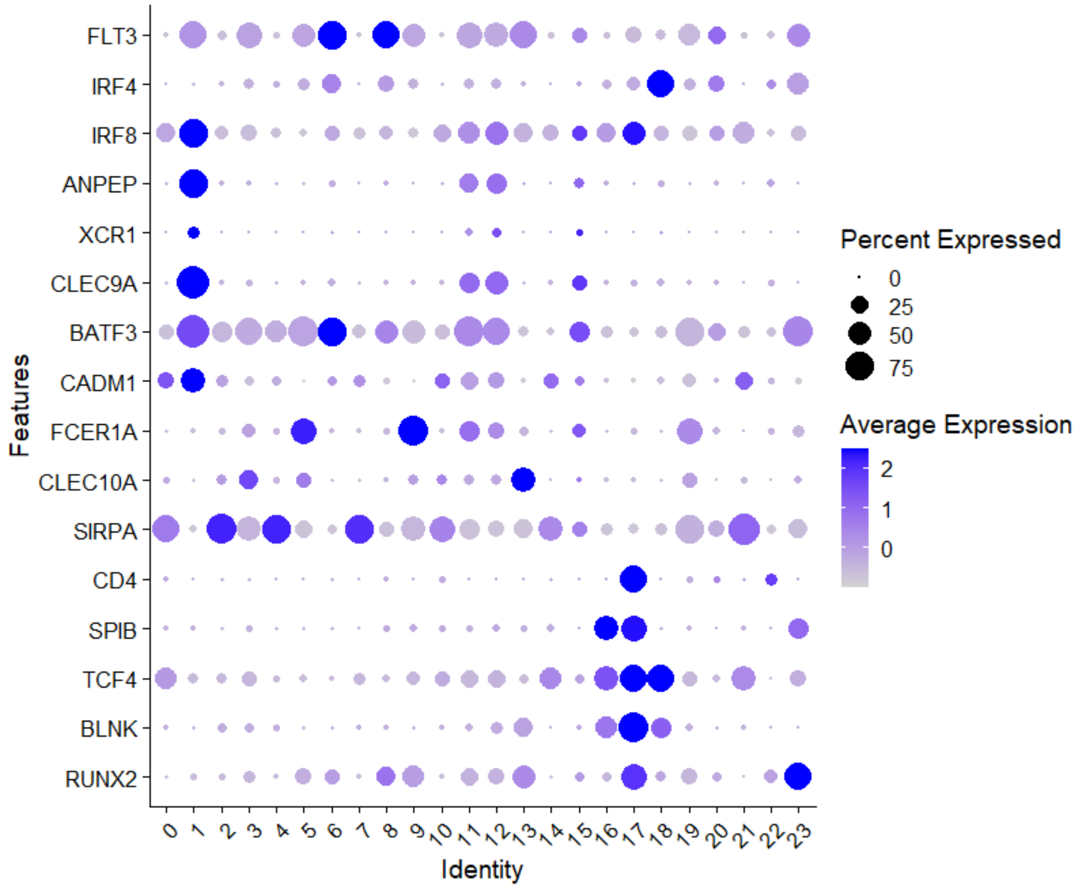

Supplementary File 5 Key-gene expression defining DC subsets visualized in a dot plot for the complete dataset.
