# Supplementary material for: Single-cell transcriptomics reveals striking heterogeneity and functional organization of dendritic and monocytic cells in the bovine mesenteric lymph node
Source: Front Immunol. 2023 Jan 6;13:1099357. doi: 10.3389/fimmu.2022.1099357 (PMC9853064; doi:10.3389/fimmu.2022.1099357)

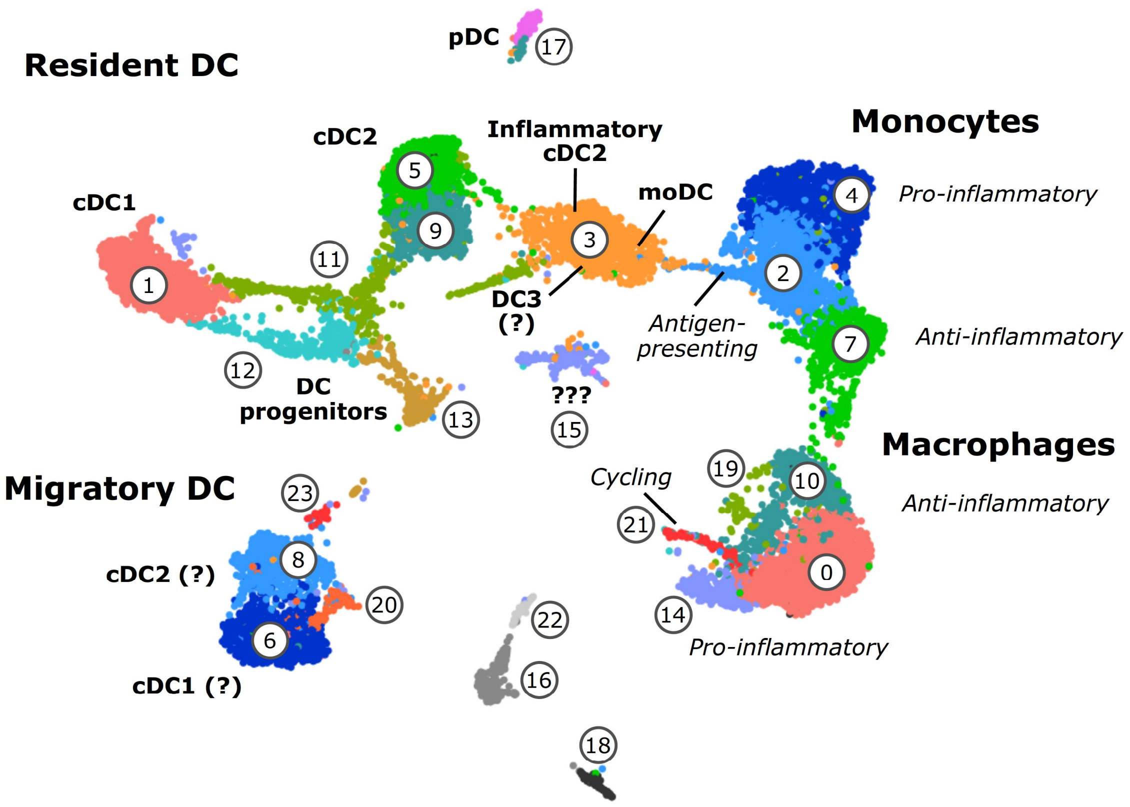

Visualization of selected genes

Page 2 | 1) Pattern-recognition receptors
2) Fc receptors
3) Purinergic receptors

Page 3 | 4) Chemokines
5) Chemokine receptors

Page 4 | 6) Integrins
7) Galectins

Page 5 | 8) Antigen presentation
9) T-cell modulation

Page 6 | 10) Interleukins
11) Interleukin receptors

Page 7 | 12) TNF
13) TNF receptors

Page 8 | 14) Tetraspanins
15) Metalloproteinases

Page 9 | 16) Metabolism (misc.)
17) Glycolysis

Page 10+11 | 18) Solute carriers

Page 12 | 19) Complement system
20) Interferon-associated

Page 13 | 21) Retinoic-acid production and signaling
22) Semaphorins and receptors

1) Pattern-recognition receptors

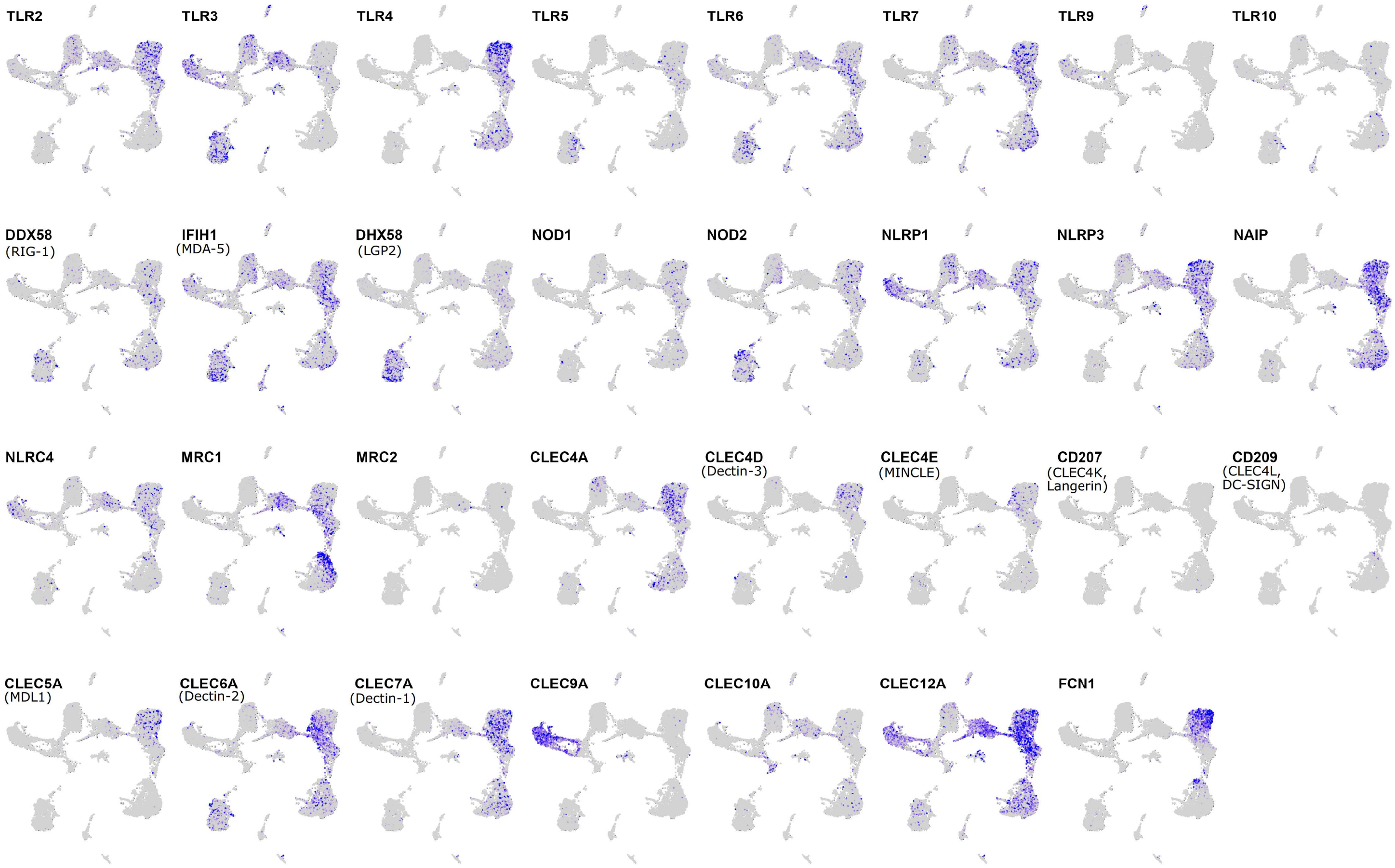

2) Fc receptors

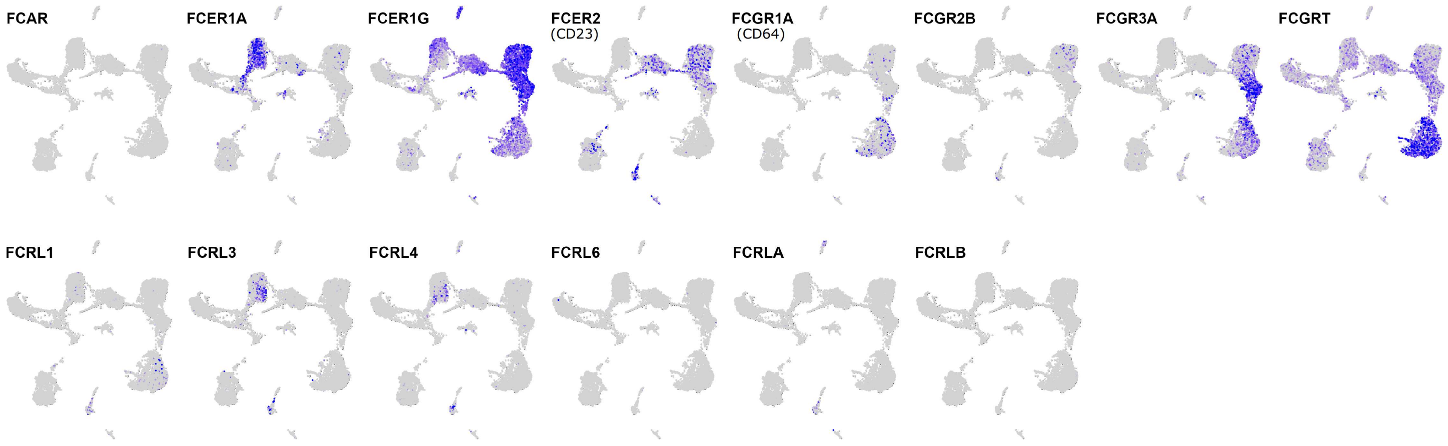

3) Purinergic receptors

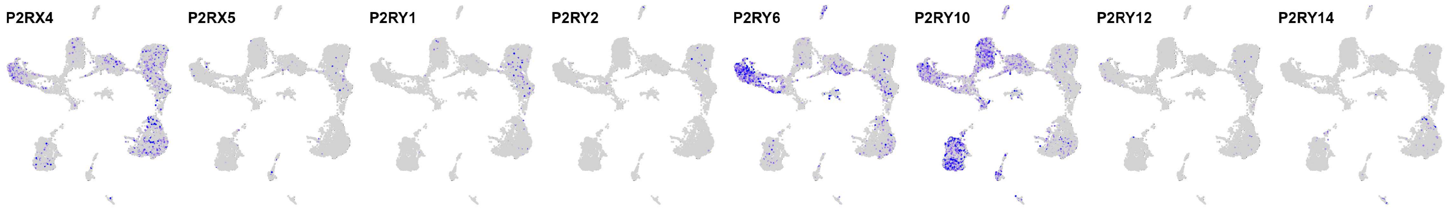

Poorly detected: P2RX1, P2RX2, P2RX3, P2RX7, P2RY8, P2RY11

4) Chemokines

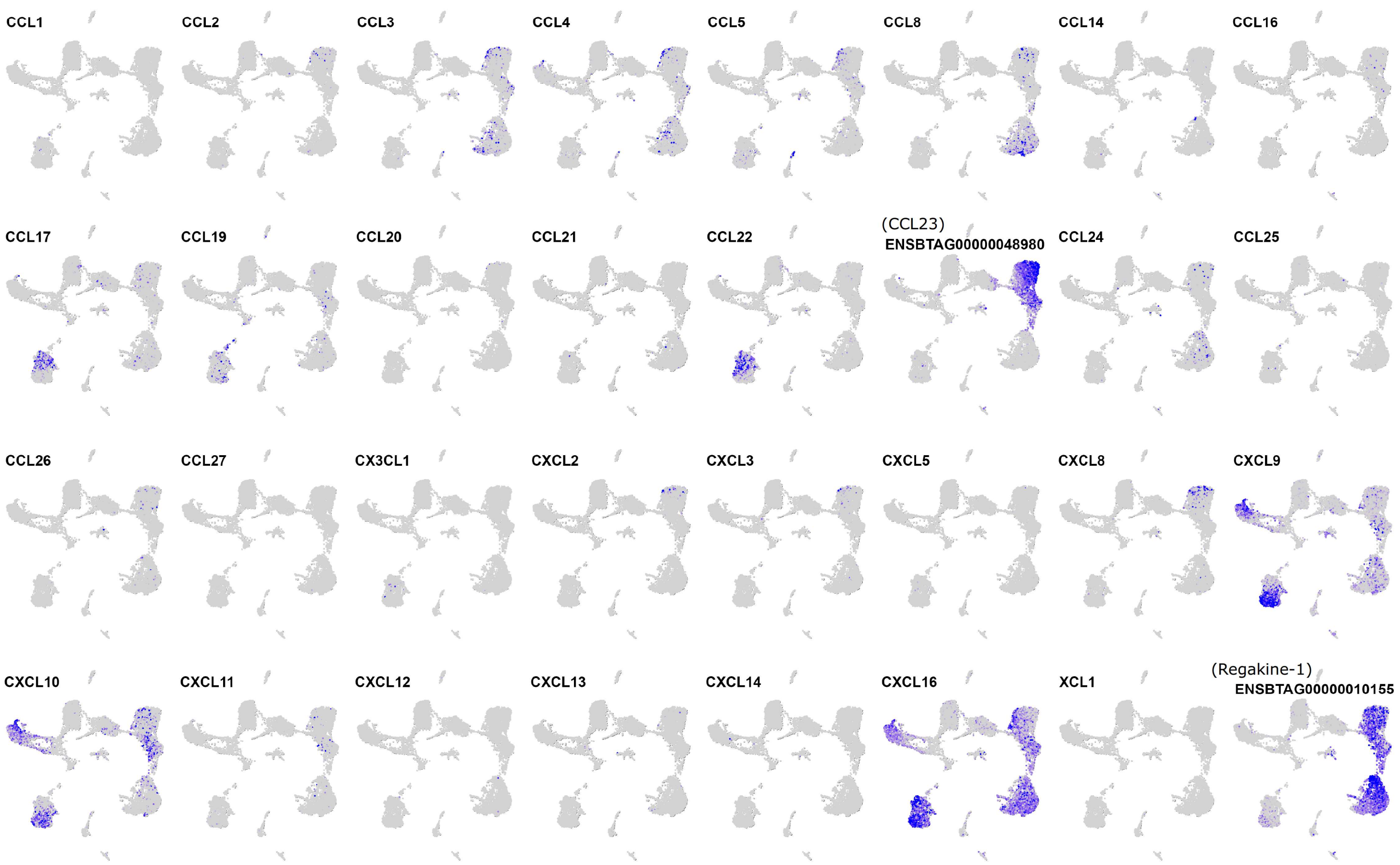

5) Chemokine receptors

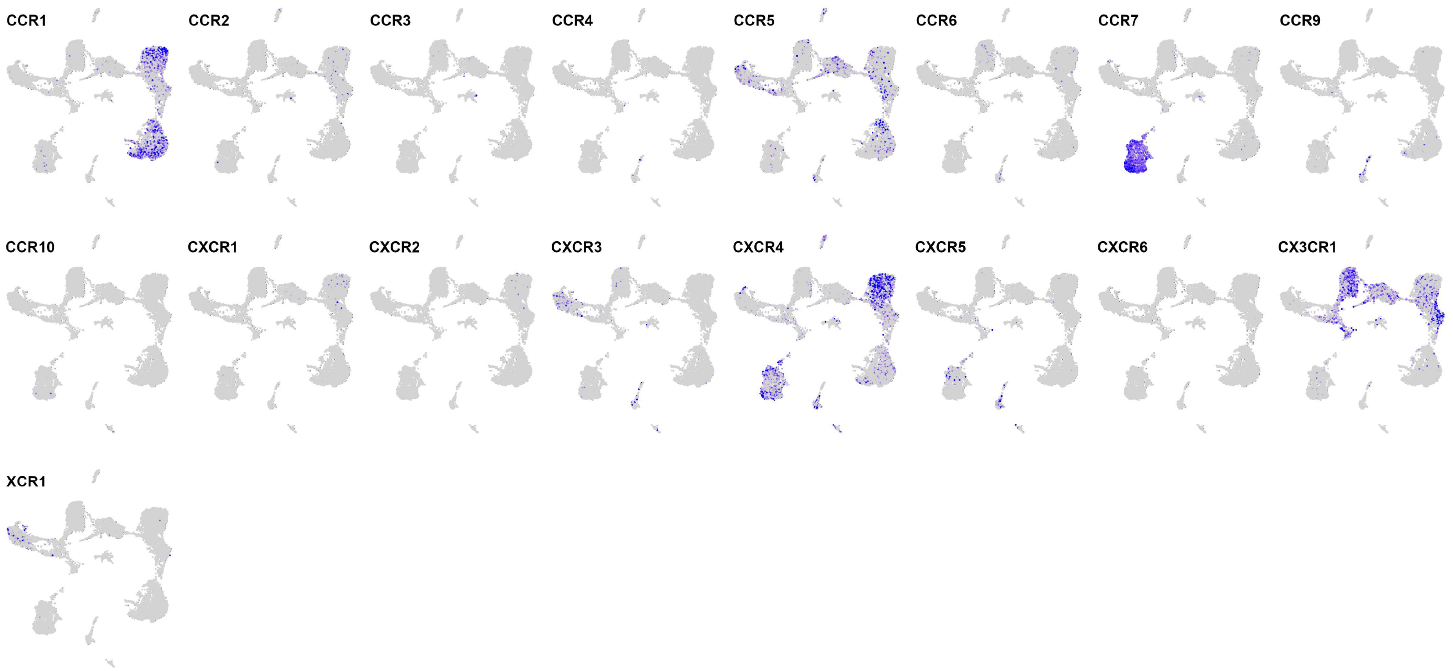

6) Integrins

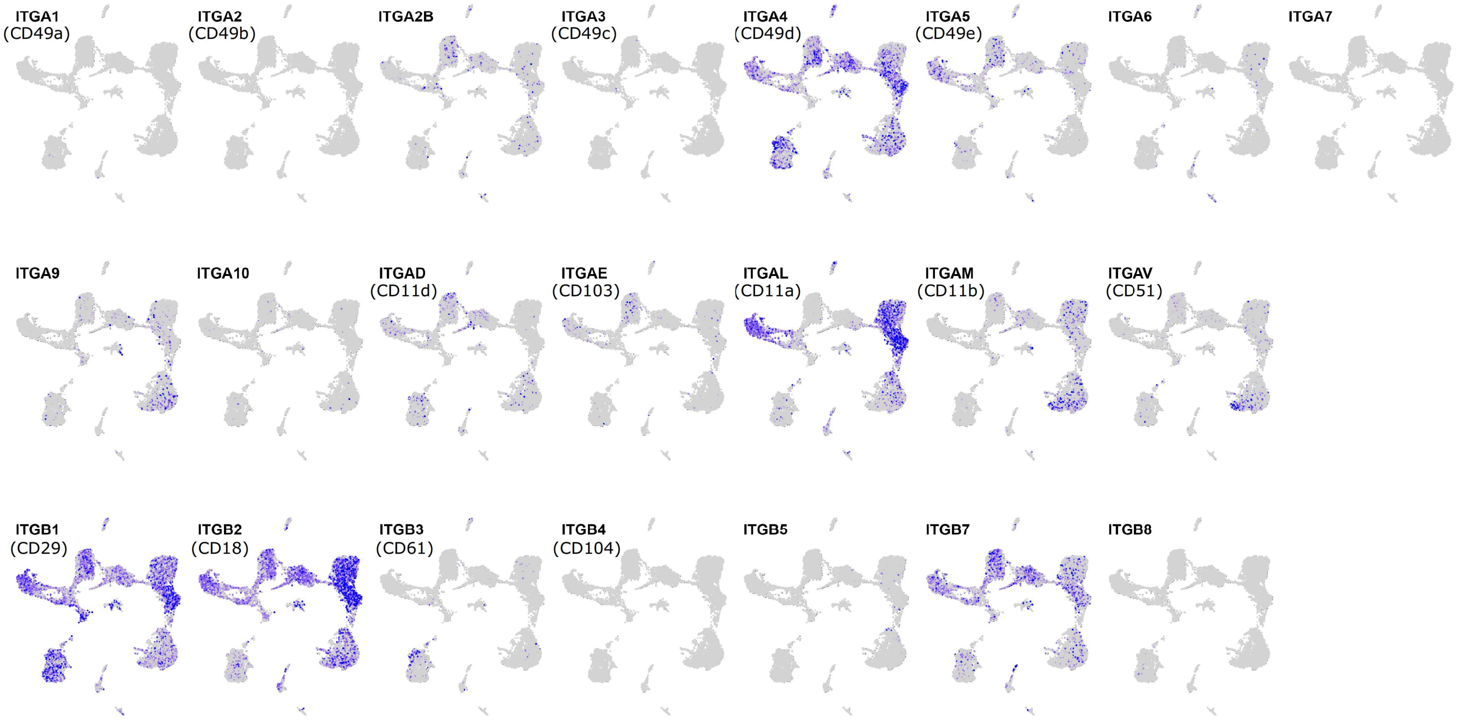

7) Galectins

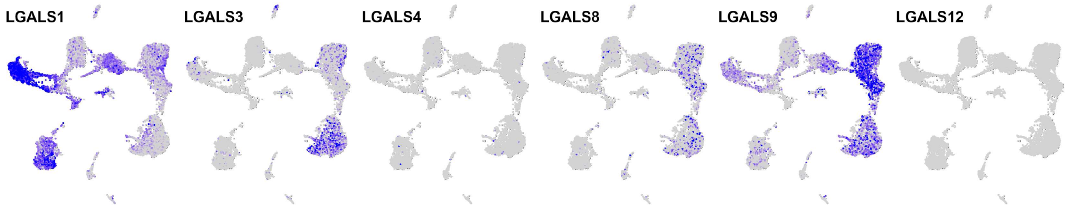

8) Antigen presentation

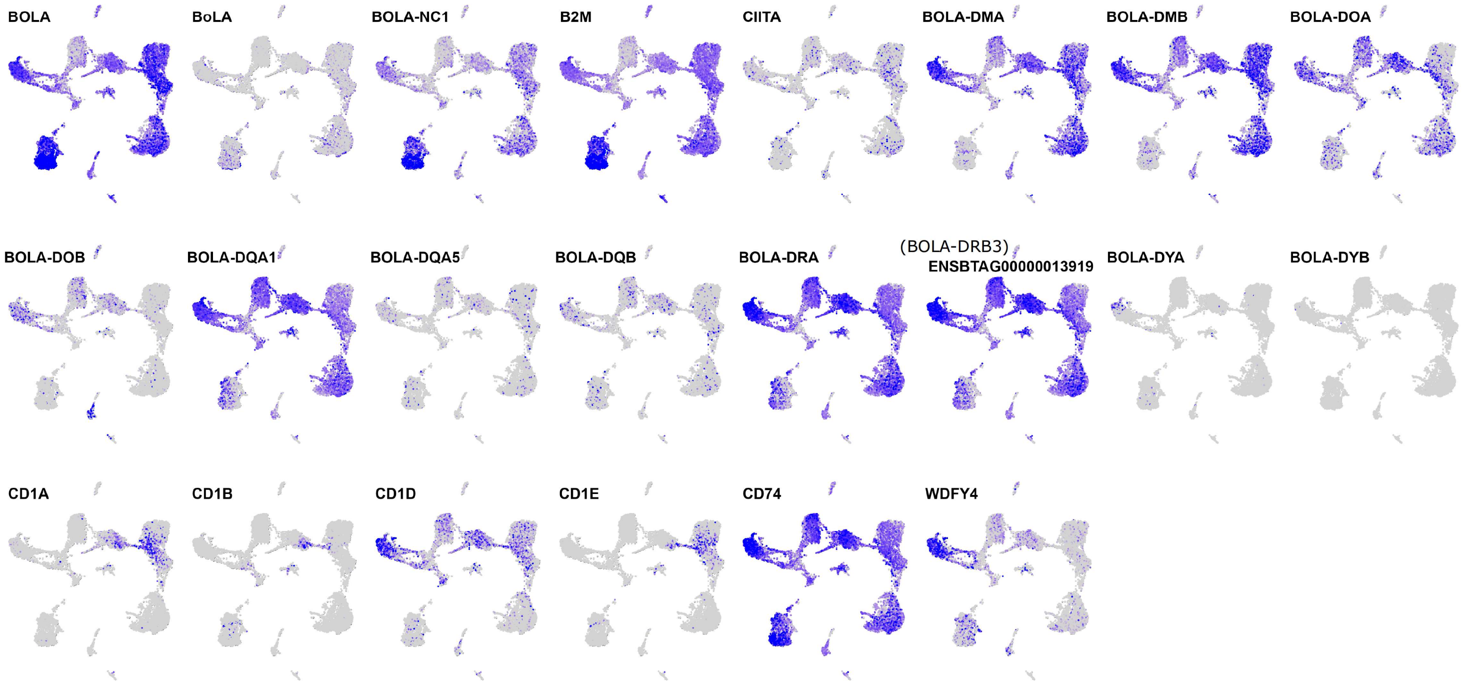

9) T-cell modulation

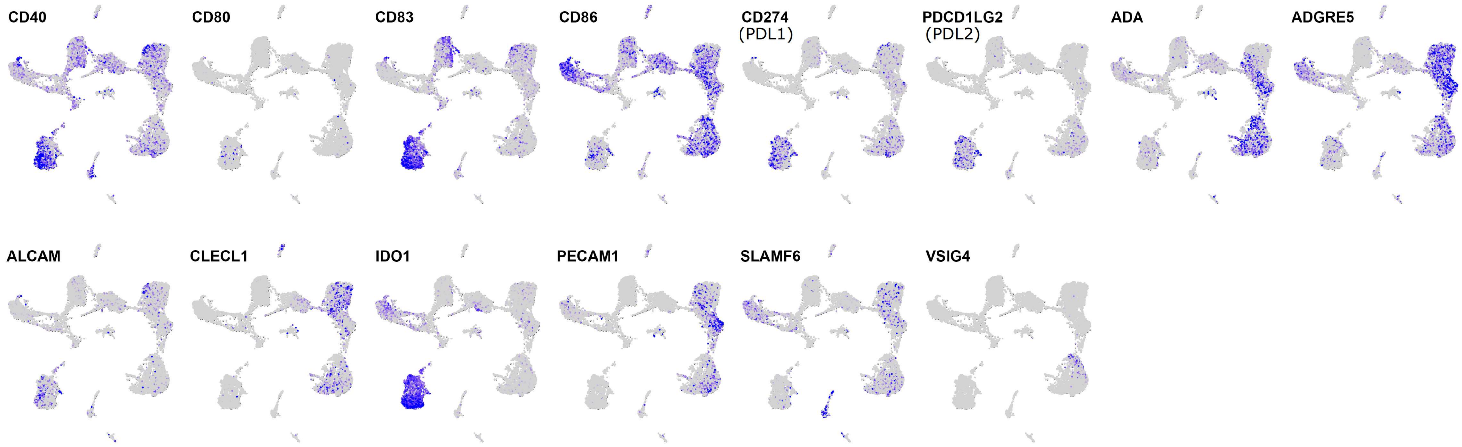

10) Interleukins

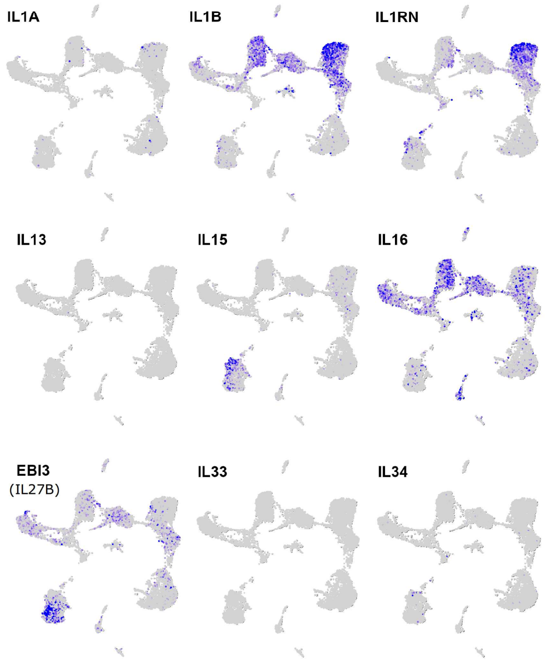

11) Interleukin receptors

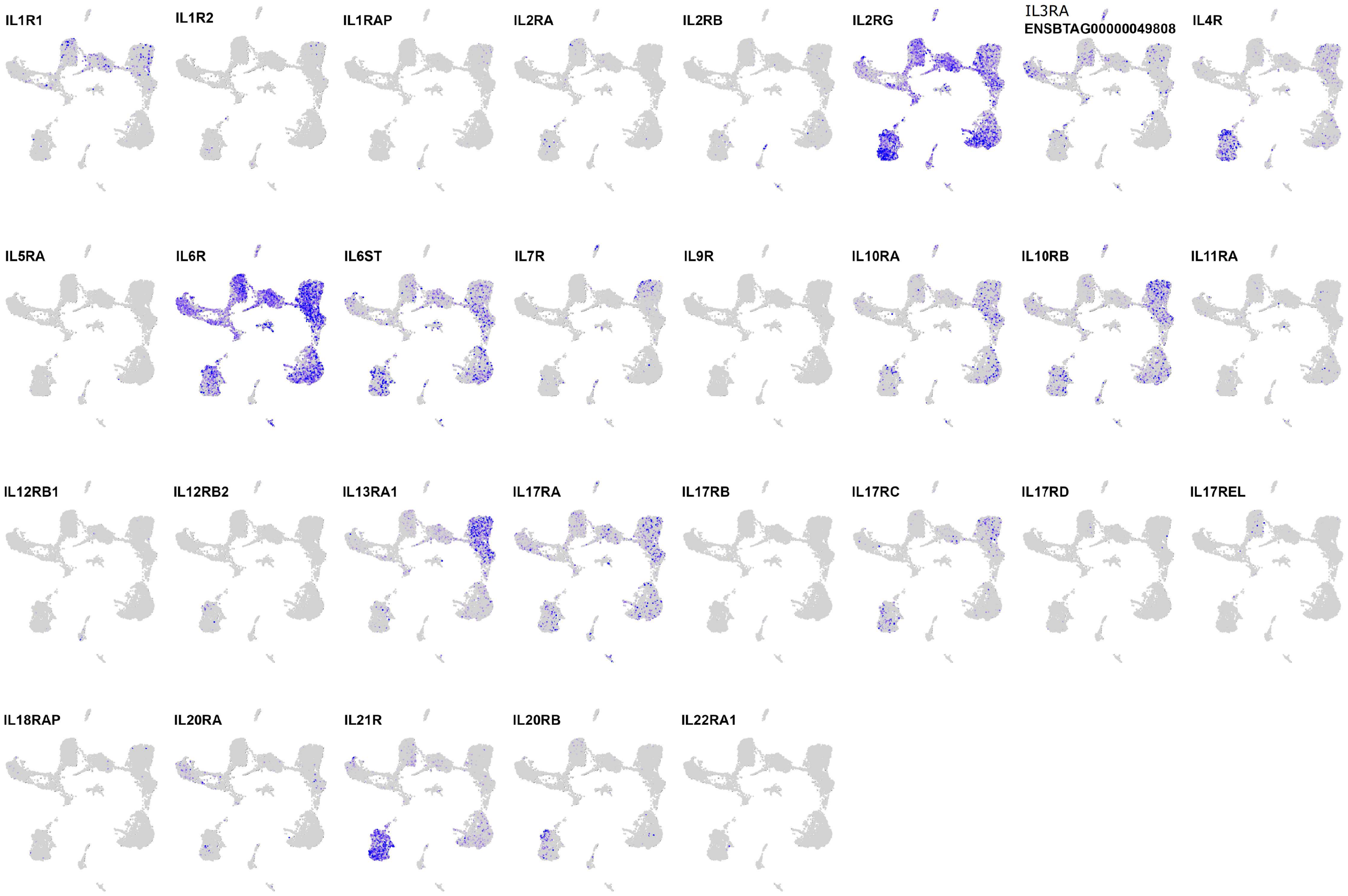

12) TNF superfamily

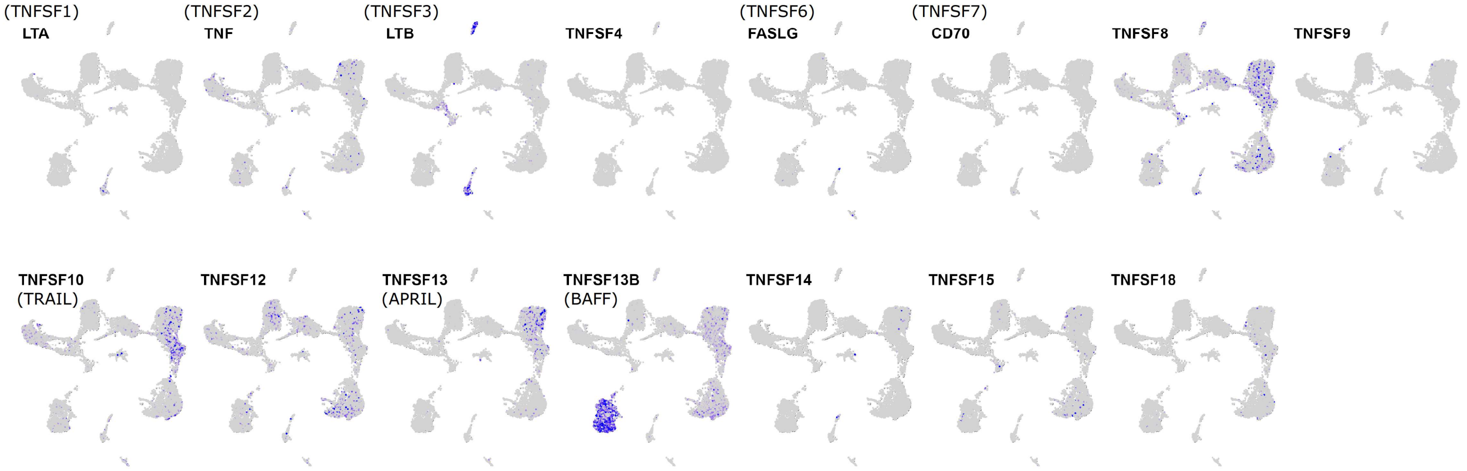

13) TNF receptor superfamily

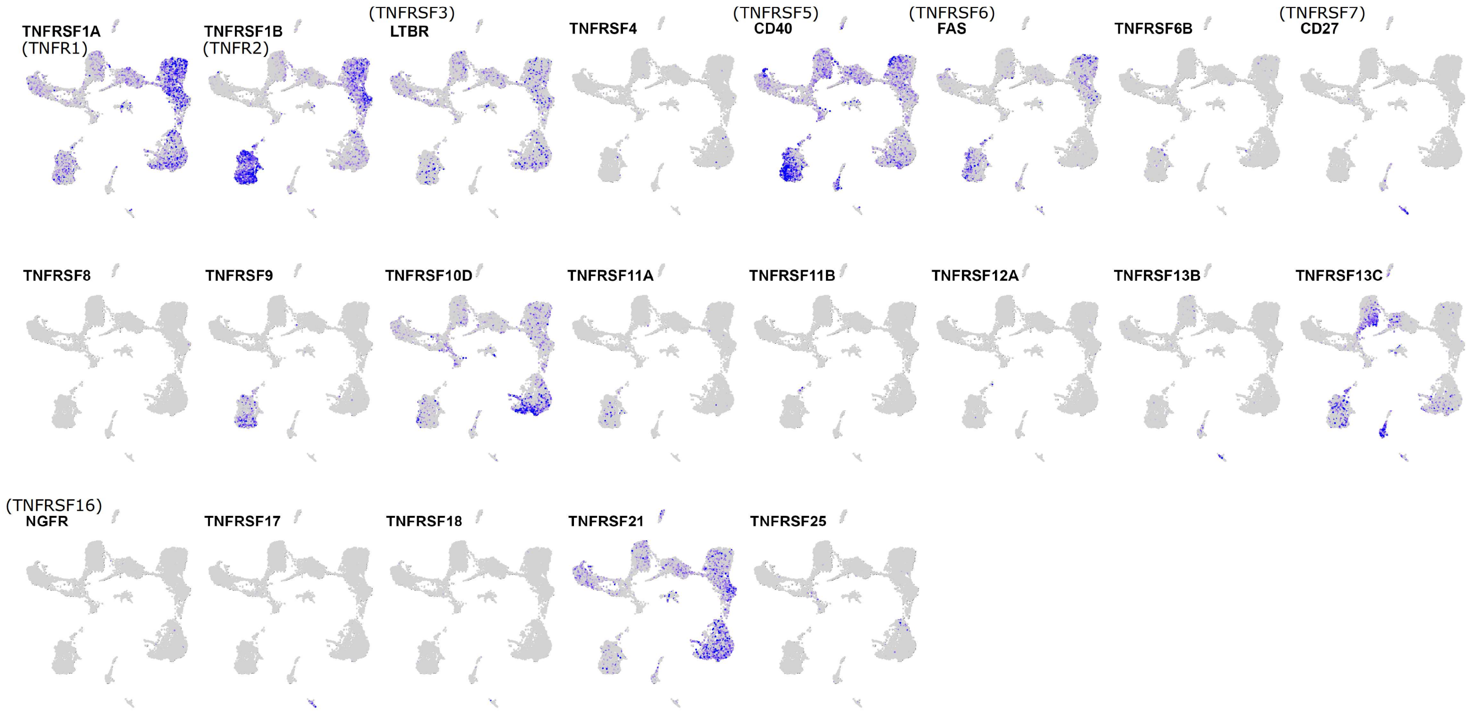

14) Tetraspanins

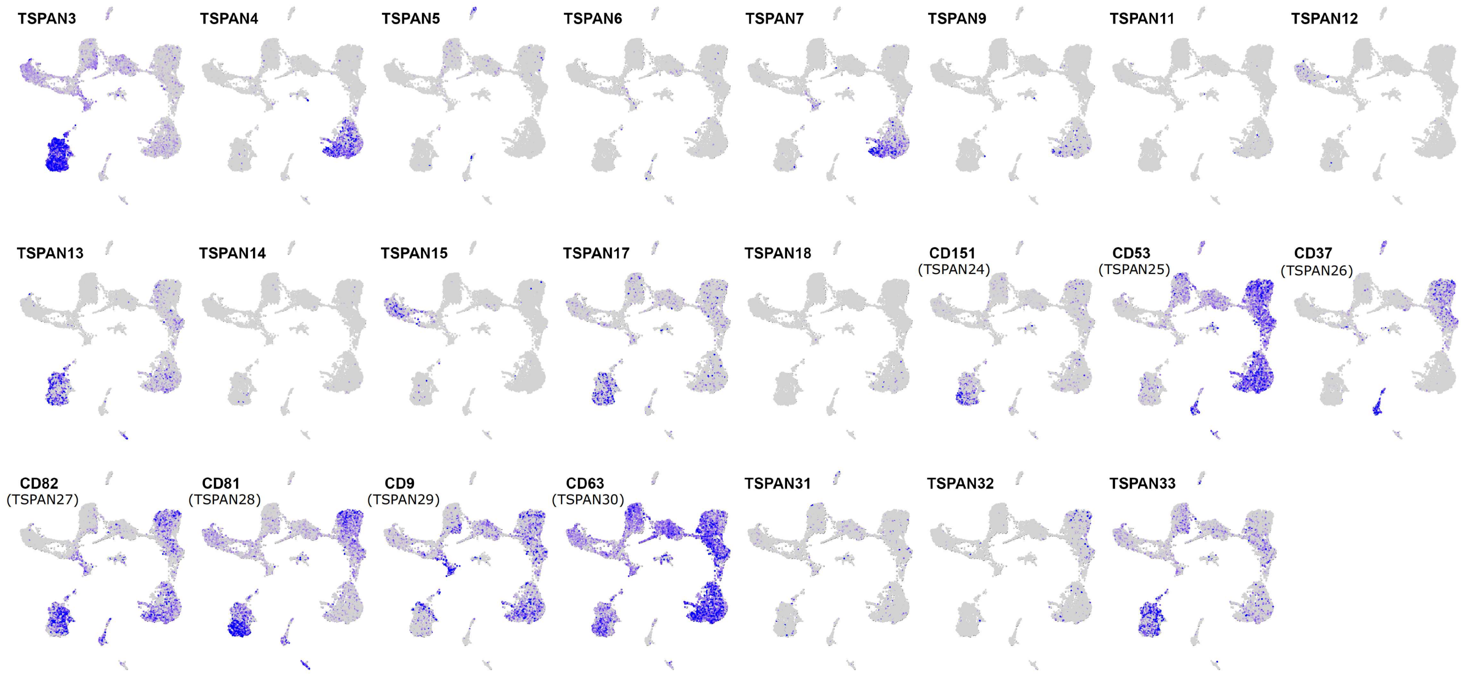

Poorly detected: TSPAN1, TSPAN2, UPK1B (TSPAN20)

15) Metalloproteinases

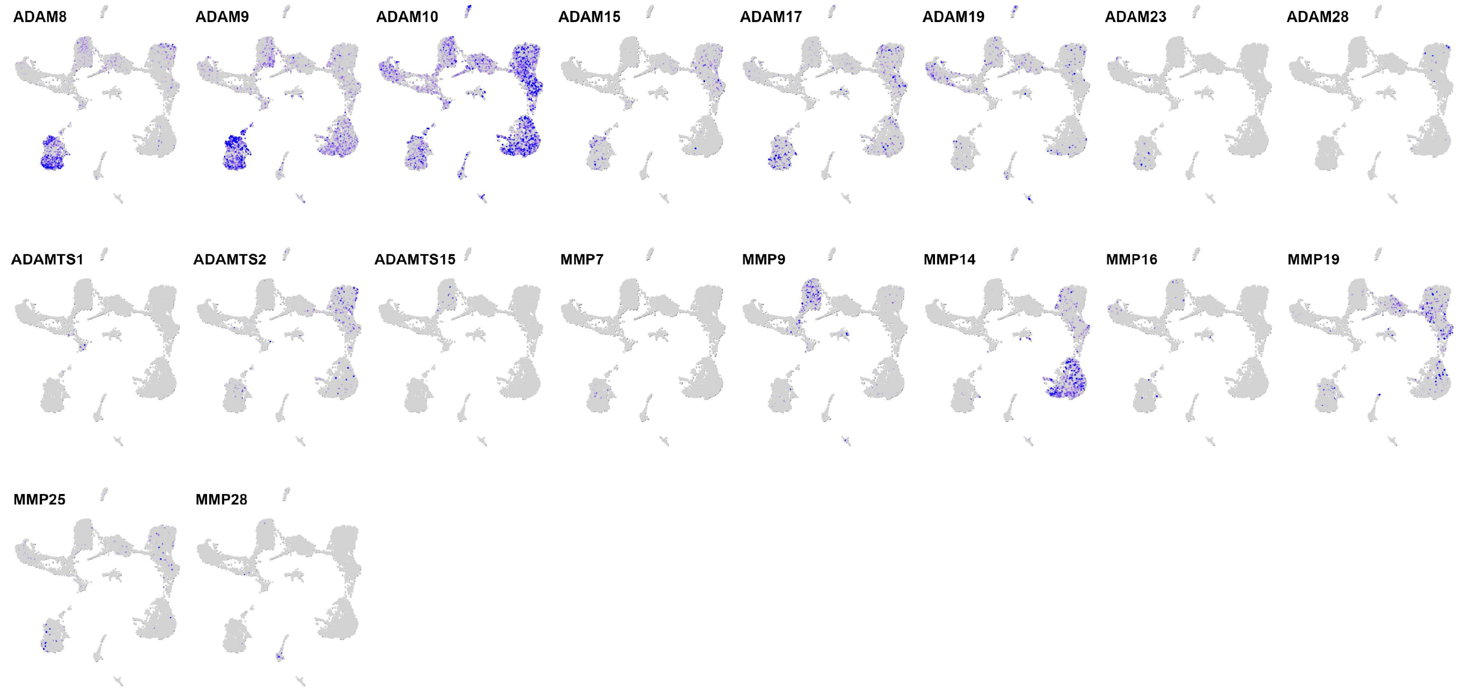

Poorly detected: ADAM11, ADAM12, ADAM20, ADAM22, ADAM32, ADAM33, ADAMTS6, ADAMTS7, ADAMTS8, ADAMTS10, ADAMTS12, ADAMTS14, MMP12, MMP15, MMP20

16) Metabolism (misc.)

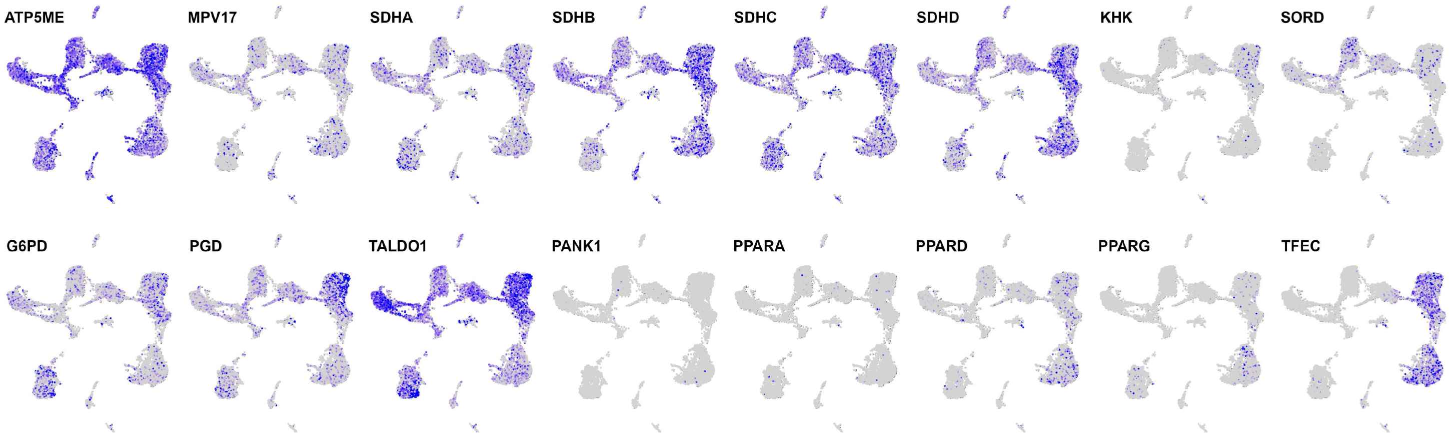

17) Glycolysis

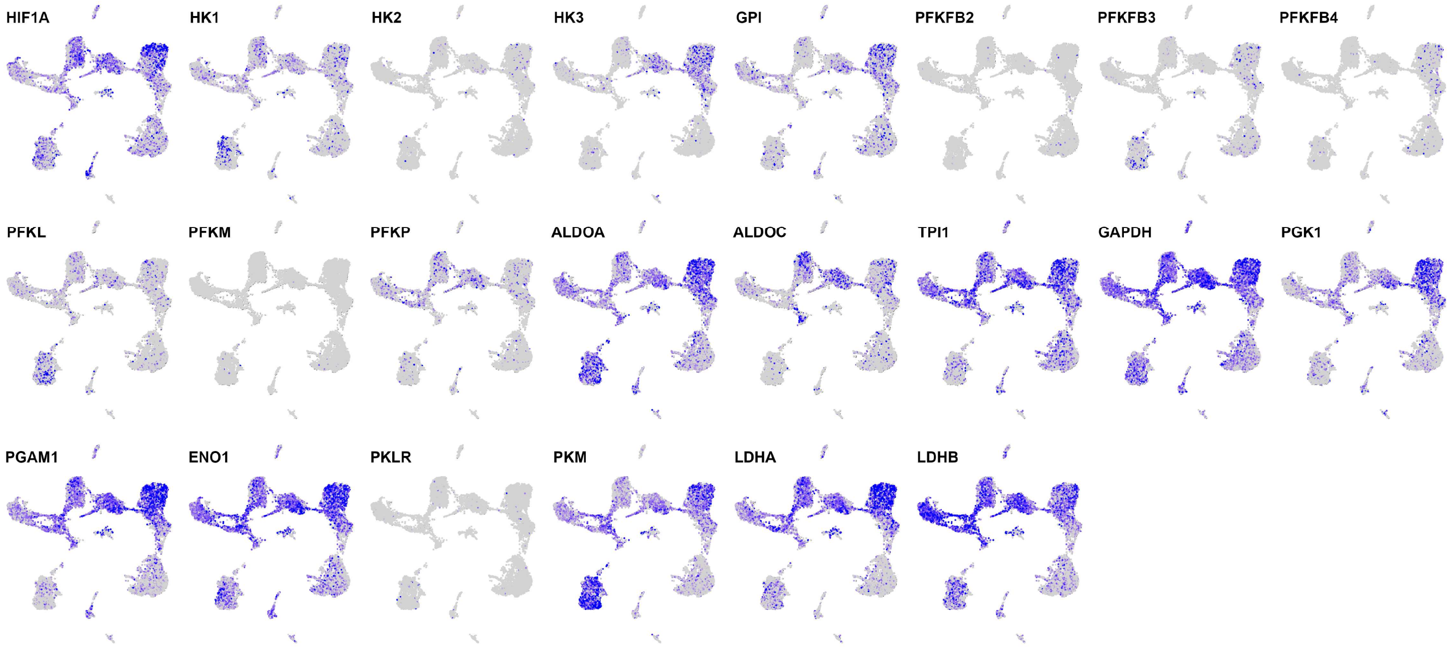

18) Solute carriers (part 1/2)

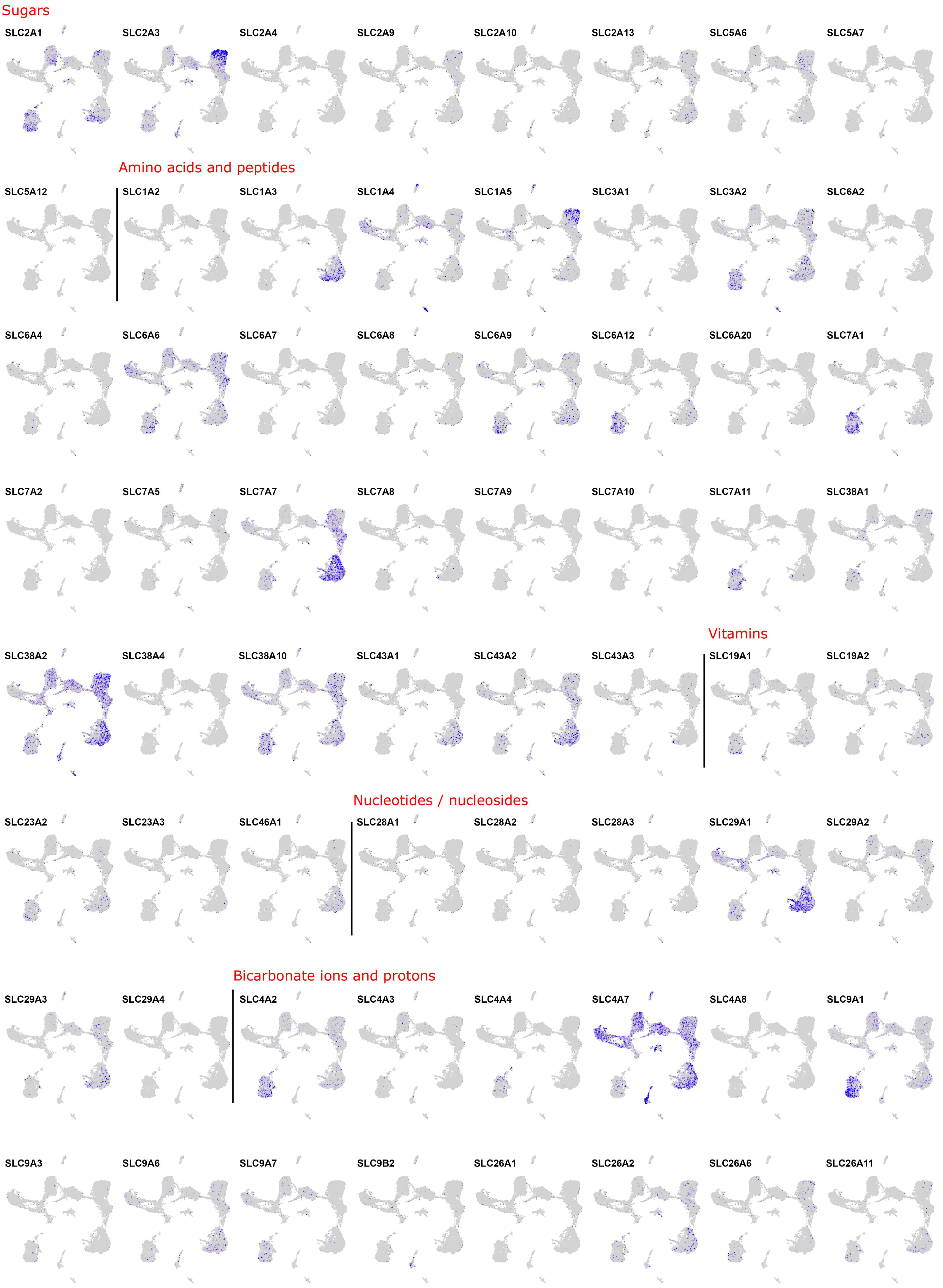

18) Solute carriers (part 2/2)

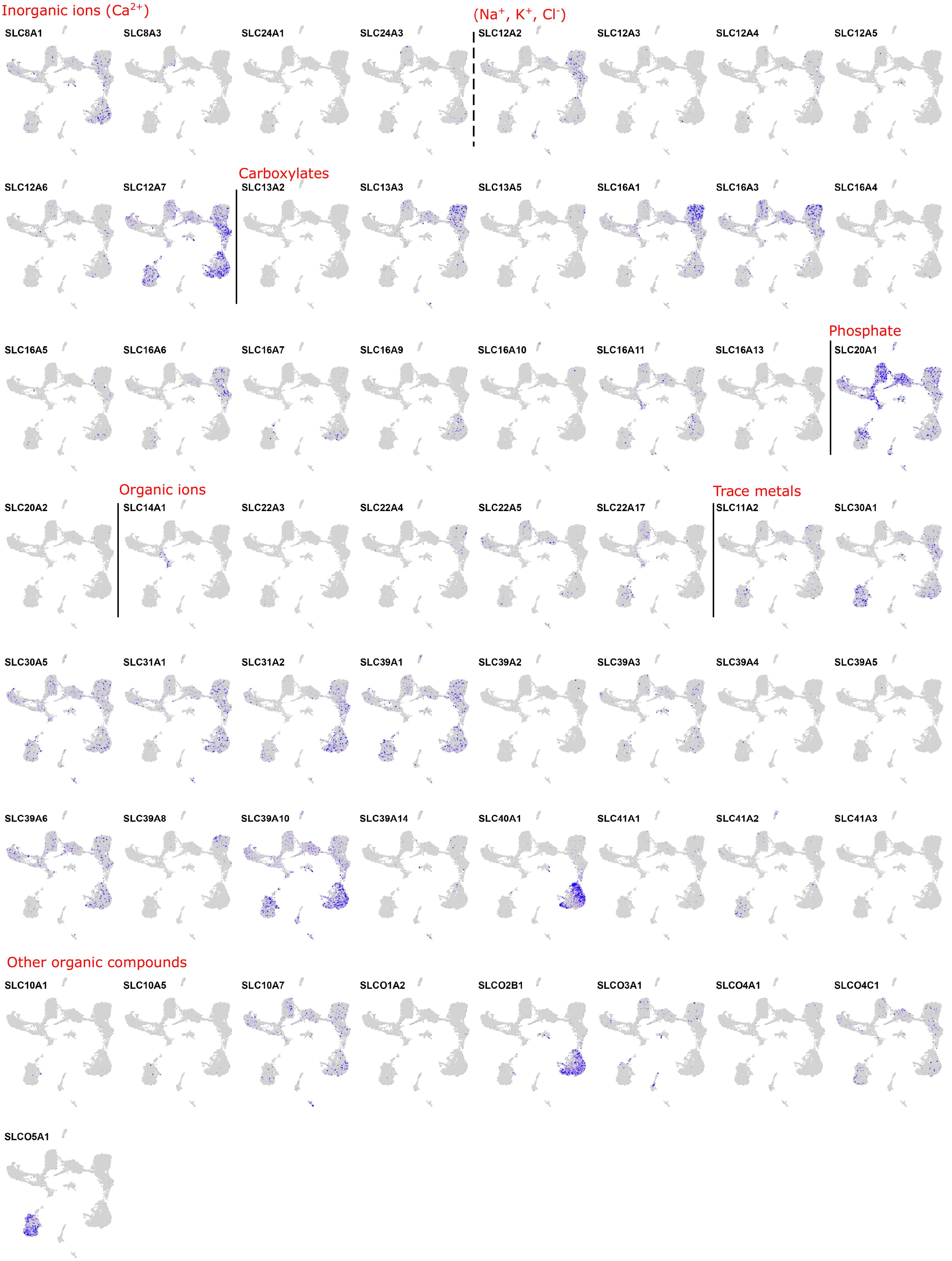

19) Complement system

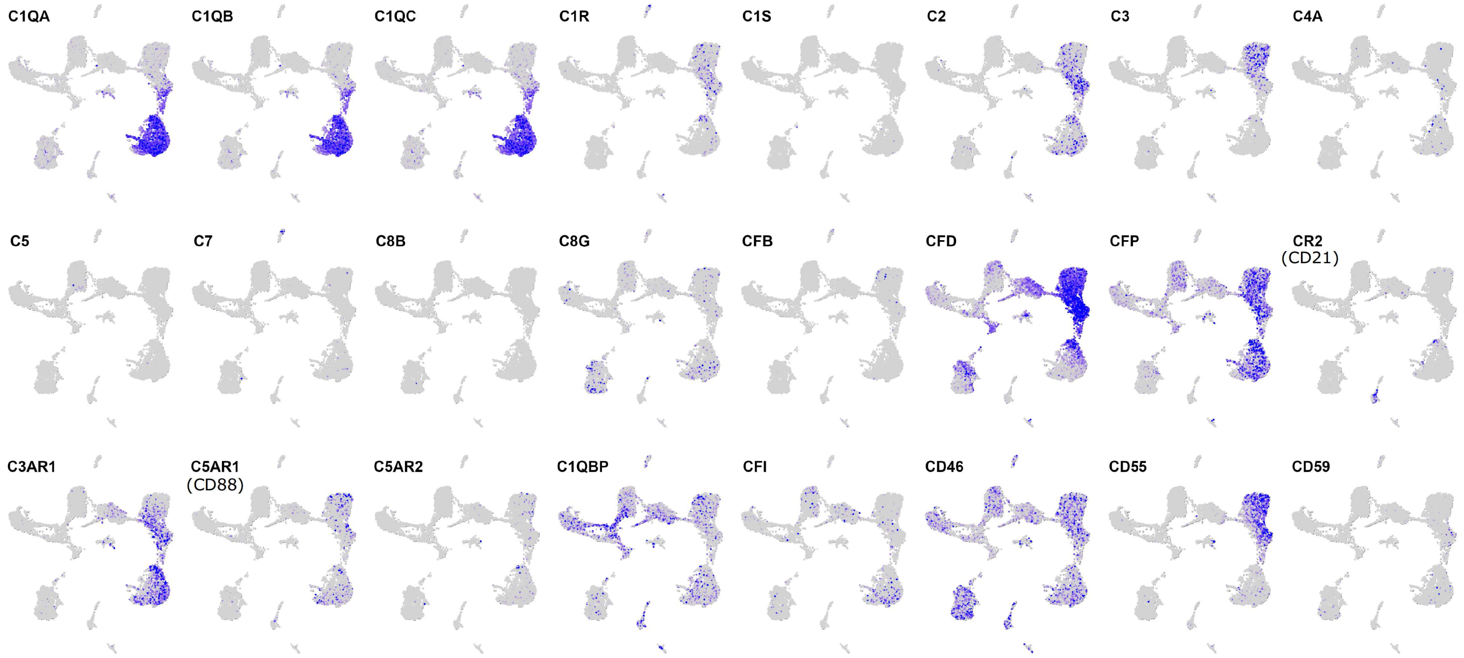

20) Interferon-associated

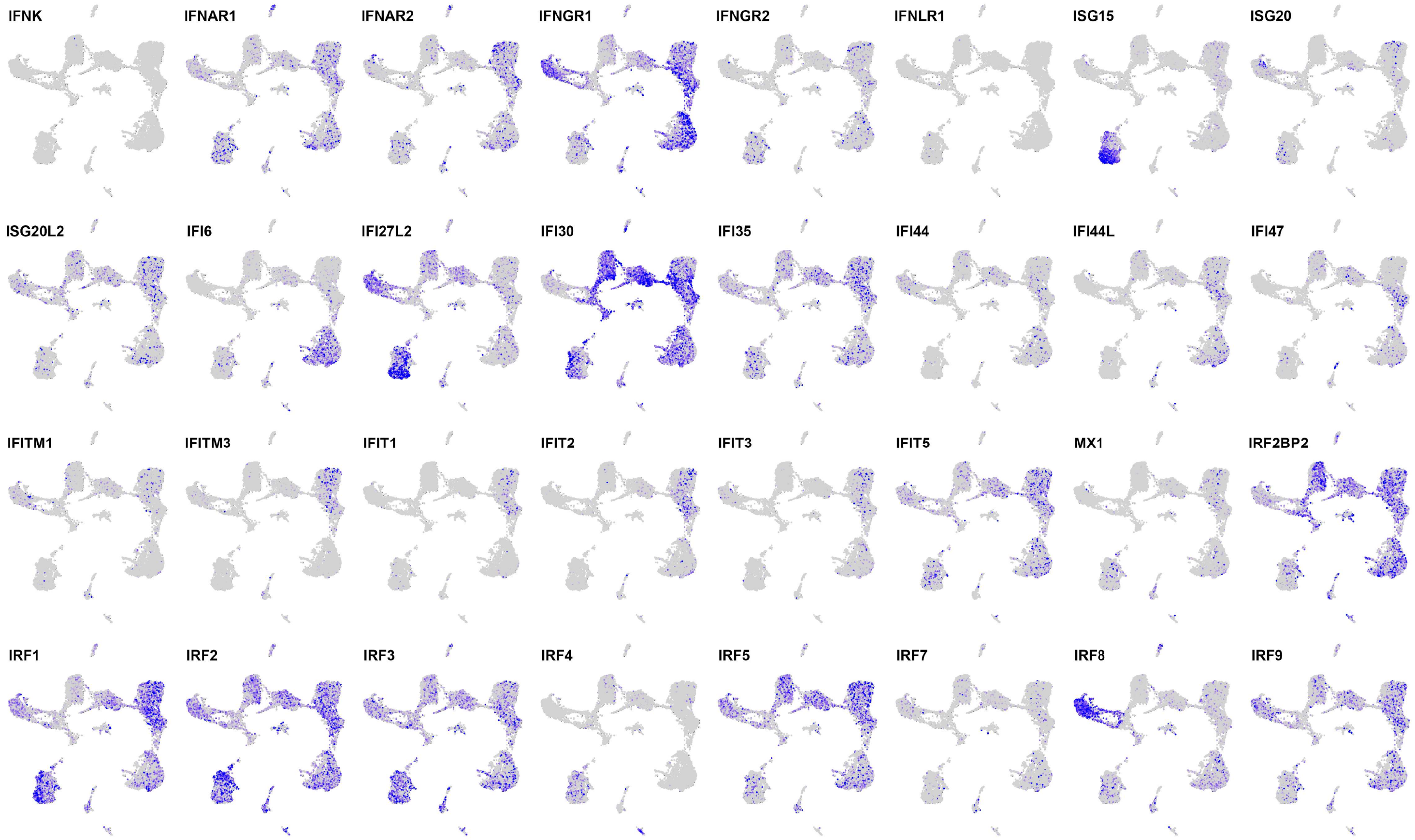

21) Retinoic-acid production and signaling

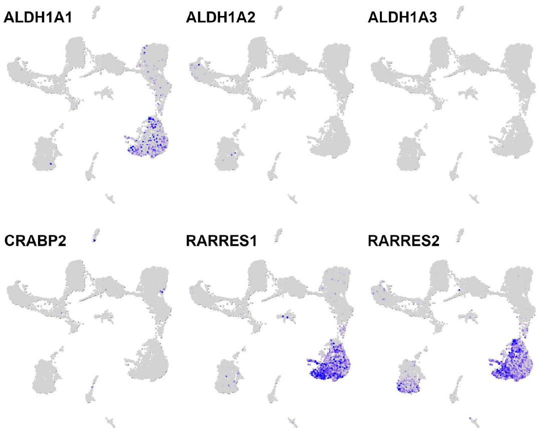

22) Semaphorins and receptors

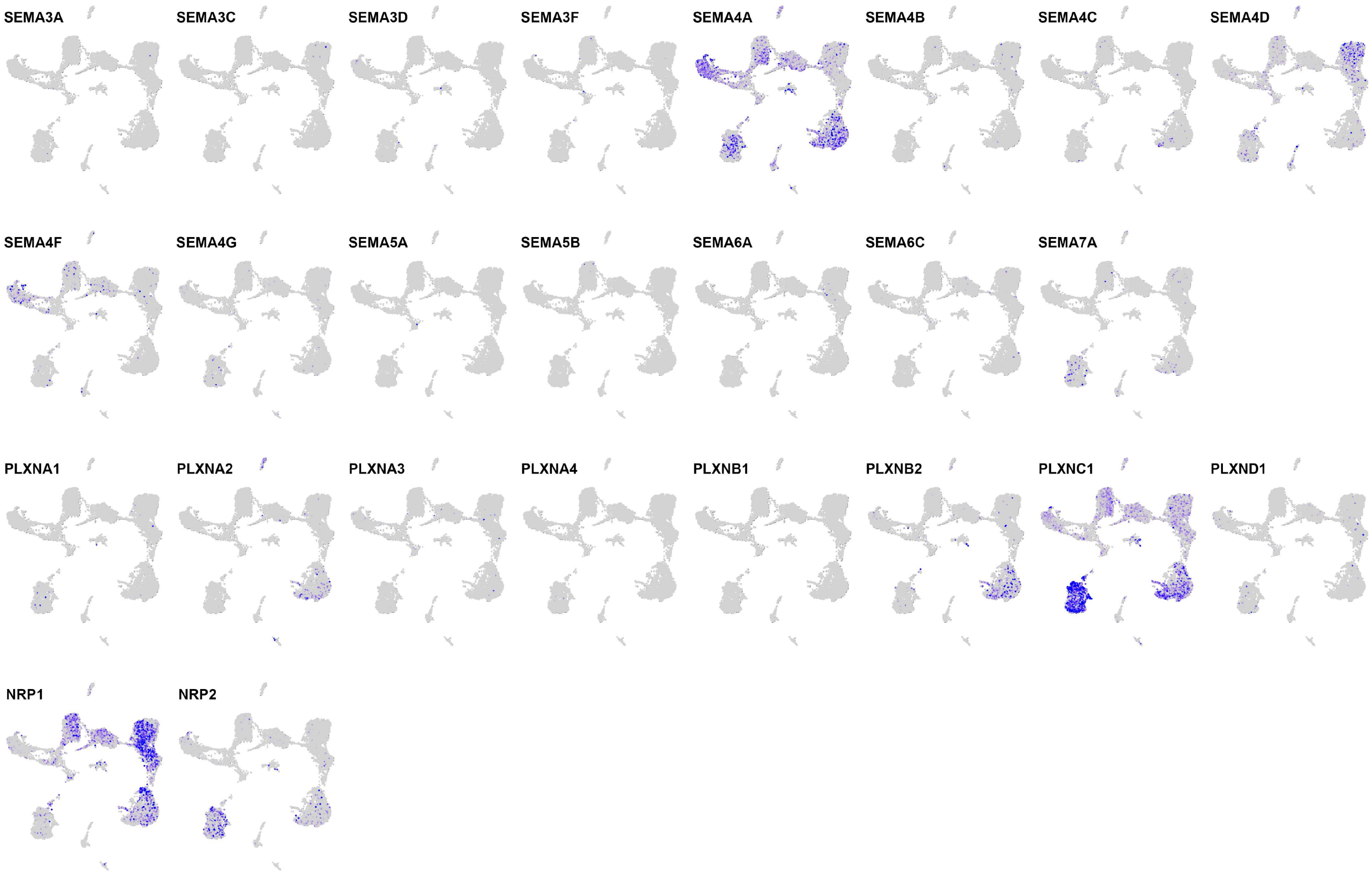

Supplement: Supplementary file 12 [file DataSheet_12.pdf]
